# Lactylation, Crotonylation and Succinylation: Decoding Their Roles in the Progression of Cardiovascular Disease

**DOI:** 10.31083/RCM45696

**Published:** 2026-02-04

**Authors:** Xuchao Hu, Yinchang Zhang, Qiming Zhao, Yongnan Li, Xiangyang Wu

**Affiliations:** ^1^Department of Cardiac Surgery, The Second Hospital of Lanzhou University, 730000 Lanzhou, Gansu, China

**Keywords:** post-translational modifications, cardiovascular diseases, crotonylation, lactonylation, succinylation

## Abstract

Cardiovascular diseases (CVDs), such as atherosclerosis, myocardial remodeling, myocardial ischemia-reperfusion (I/R) injury, heart failure, and oxidative stress, are among the greatest threats to human health globally. The molecular mechanisms underlying CVDs have not yet been fully elucidated, but progress has been made in research on epigenetics in CVDs. Post-translational modifications (PTMs), which involve the covalent attachment of functional groups to modulate protein structure and function, represent a critical regulatory mechanism. These modifications enhance the functional diversity of the proteome without the need for de novo protein synthesis. Traditional types of PTMs, such as phosphorylation, acetylation, and ubiquitination, are closely associated with the pathogenesis of CVDs. With the application of high-performance liquid chromatography-tandem mass spectrometry (HPLC-MS/MS), an increasing number of novel acylation modifications have been discovered, including propionylation, butylation, crotonylation, succinylation, lactylation, and isonicotinylation. A deeper understanding of the role of PTMs in CVDs is essential for unraveling their molecular regulatory mechanisms and identifying new biomarkers and therapeutic targets. This review summarizes the mechanisms related to the occurrence and development of CVDs associated with three novel acylation modifications: crotonylation, lactylation, and succinylation.

## 1. Introduction

Cardiovascular diseases (CVDs) have become one of the major causes of global diseases. Researchers characterize CVDs by their increased incidence, prevalence, recurrence, mortality rates, and substantial economic burden. Globally, both morbidity and mortality of CVDs have shown a significant upward trend over the past decades [1]. From 1990 to 2019, the number of global CVD cases surged from 270 million to 523 million, almost doubling. Simultaneously, the number of deaths caused by CVDs has also continued to grow, steadily increasing from 12.1 million in 1990 to 18.6 million in 2019 [2]. These data highlight the urgency and importance of prevention and control of CVD globally.

The epidemiologic background of CVDs highlights their status as a significant threat to both global health and those of the Chinese population. Their increased prevalence, high mortality rates, and heavy disease burden create an urgent need to strengthen preventive, control, and therapeutic measures to improve the epidemiology of CVDs and safeguard human health. The occurrence and development of CVDs are closely related to the environment and patients’ behaviors. Although the causative factors may differ, the main mechanisms of CVD are related to signal transduction pathways and the supply and capacity of oxygen in the mitochondria. In recent years, epigenetic modifications such as DNA methylation, histone modification, histone acetylation, and RNA interference have been involved in the pathophysiology of CVDs [3]. Post-translational modification of proteins involves chemical modification of specific amino acid residues, a widespread phenomenon that plays an indispensable role in mammalian cells and, in particular, can regulate cellular molecular functions. Post-translational modifications (PTMs) can act as reversible functional regulators of eukaryotic cells. Metabolic enzymes regulate intracellular metabolite levels to support PTMs. One of the standard characteristics of human diseases is that they are affected by aberrant post-translational regulation of proteins *in vivo*. In CVDs, PTMs of proteins are crucial. They not only affect the structure and function of proteins but also participate in regulating normal physiological activities of the cardiovascular system. When PTMs are abnormal, they may lead to the occurrence and development of CVDs. Therefore, an in-depth study of the mechanism of PTMs in CVDs is of great significance in the search for new therapeutic approaches and drug interventions. In this paper, we review the physiological and pathological effects of crotonylation, lactylation, and succinylation on CVD and the research progress that has been made in recent years worldwide.

## 2. Crotonylation

Histone lysine crotonylation (Kcr) is a newly discovered protein post-translational modification (PTM) first reported by Tan *et al*. in 2011 [4]. This modification involves transferring the crotonyl group on crotonyl coenzyme A to lysine residues, a reaction that requires histone crotonyltransferase (HCT) [5]. Crotonylation is a regulated reversible modification process, in addition to crotonyltransferases by decrotonylase, with opposite enzymatic activity. The terms “writer”, “eraser”, and “reader” summarize this modification process. “Writer” refers to the crotonyltransferase, which is responsible for catalyzing the crotonylation of lysine residues; “eraser” refers to the decrotonylase, which is responsible for removing the crotonyl group from lysine residues; and “reader” refers to the enzyme that is responsible for removing the crotonyl group from lysine residues. These terms refer to several proteins that specifically recognize this modification and can convert it to various cellular functions. Histone lysine crotonylation modification can perform multiple tasks because it can recruit downstream reader proteins. In addition, non-histone proteins have also been found to undergo a crotonylation modification [6]. Although Kcr shares common properties with acetylation regarding certain regulatory factors and sites, the unique C-C-π bond structure of the crotonyl group confers Kcr with its unique biological properties [7, 8].

Due to the importance of crotonylation modifications, many research teams are actively investigating their specific mechanisms of action within the cell. Understanding how Kcr modification affects protein-protein interactions, how intracellular reader proteins recognize it, and its role in signaling pathways is essential to unravel its biological functions. Further research shows that histone crotonylation can trigger gene transcription and regulate metabolism, DNA repair, depression, and reproductive development [9]. In this review, we present the regulation and effects of crotonylation modifications on CVDs from both a cardiomyocyte and non-cardiomyocyte perspective.

### 2.1 Cardiomyocytes

**Fig. 1.**
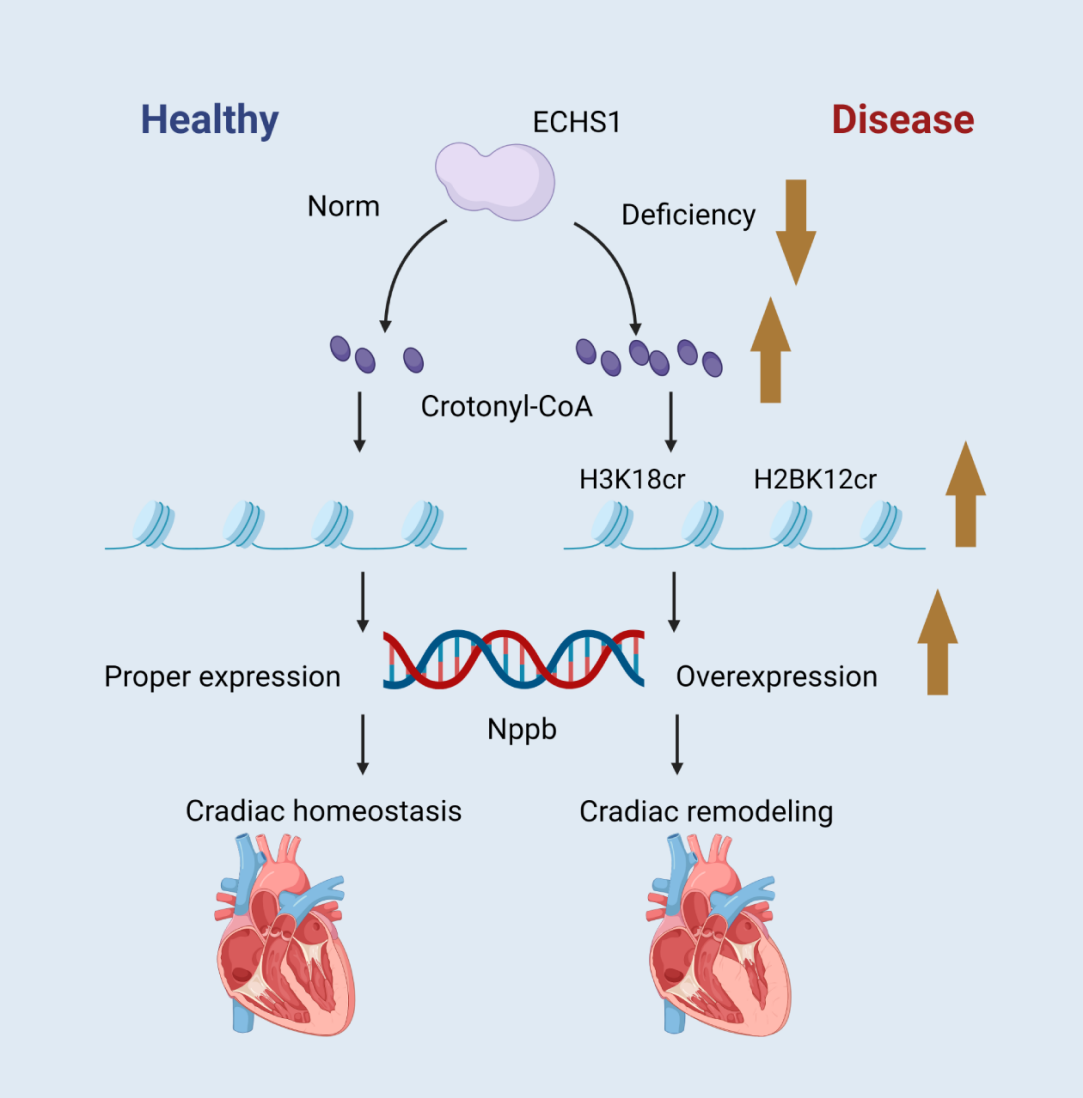
**ECHS1 controls the intracellular crotonyl-CoA and maintains the maturity and homeostasis of cardiomyocytes via histone crotonylation**. Schematic created by the authors. ECHS1, short-chain enoyl coenzyme A hydratase. The brown arrow indicates an increase or decrease in the substance next to it, up for an increase and down for a decrease. Figure created by BioRender.

A study found that short-chain enoyl coenzyme A hydratase (ECHS1) was downregulated, and H3K18cr and H2BK12cr were up-regulated in the hearts of patients with hypertrophic cardiomyopathy (Fig. 1) [10]. ECHS1 is a metabolizing enzyme whose primary function is to hydrolyze crotonoyl coenzyme A, thereby regulating intracellular crotonoyl coenzyme A levels. Crotonyl coenzyme A is a donor for the crotonylation modification of histones, and ECHS1 reduces the level of crotonylation modification of histones by decreasing the intracellular concentration of crotonyl coenzyme A, which in turn reduces the level of crotonylation modification of histones. ECHS1 hydrolyzes crotonyl coenzyme A to short-chain hydroxyl acyl-coenzyme A, reducing the amount of crotonyl coenzyme A available for histone crotonylation, thereby affecting gene expression and cellular function. The regulation of histone crotonylation modification by ECHS1 affects gene expression and cell signaling. In gene expression regulation, histone crotonylation modification promotes gene transcription. ECHS1 inhibits gene expression associated with cardiac hypertrophy by decreasing histone crotonylation levels. For example, overexpression of ECHS1 inhibits the expression of the Nppb (brain natriuretic peptide precursor B) gene mediated by crotonylation modification. In contrast, deletion of ECHS1 leads to an increase in Nppb gene expression. The regulatory role of ECHS1 also involves the activity of signaling factors. For example, deletion or increased crotonylation modification of ECHS1 promotes the enrichment of nuclear factor-activated T-cell c3 (NFATc3) in the promoter region of the Nppb gene, which enhances the transcription of the Nppb gene and the development of cardiac hypertrophy [11].

ECHS1 has a regulatory role in CVDs, especially in cardiac hypertrophy. The expression level of ECHS1 is down-regulated in cardiac tissues of patients with cardiac hypertrophy, which correlates with the up-regulation of histone H3K18cr and H2BK12cr. The down-regulation of ECHS1 leads to an increase in the modification of histone crotonylation, which promotes the expression of genes related to cardiac hypertrophy and contributes to cardiac hypertrophy and structural remodeling. Its normal function contributes to the maturation and functional maintenance of cardiomyocytes, prevents cardiomyocytes from over-hypertrophy and dysfunction in response to pathological stimuli, and protects the heart from overload and injury. In neonatal congenital heart disease, mutations in the ECHS1 gene can lead to cardiomyopathy [12]. ECHS1 and its regulated histone crotonylation modification may be a potential therapeutic target for CVDs such as cardiac hypertrophy. Regulating the expression or activity of ECHS1 or intervening in the histone crotonylation modification pathway may provide new strategies for treating cardiac hypertrophy. For example, developing drugs that enhance ECHS1 activity or inhibit histone crotonylation modification may help attenuate the pathological process of cardiac hypertrophy and improve cardiac function.

**Fig. 2.**
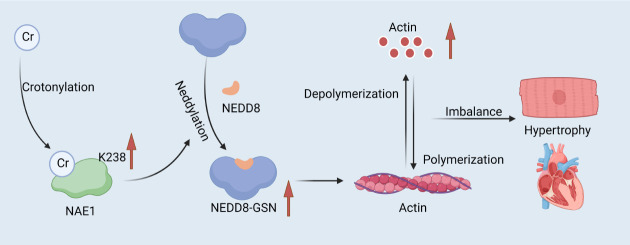
**NAE1 K238 crotonylation regulates cytoskeletal remodeling and cardiac hypertrophy by targeting GSN**. Under hypertrophic stimuli, the K238 crotonylation of NAE1 is enhanced, causing increased GSN neddylation. This process increases GSN’s protein stability and actin—severing activity. In turn, this leads to adverse cytoskeletal remodeling and cardiac hypertrophy. Schematic created by the authors. NAE1, NEDD8-activating enzyme E1 regulatory subunit; GSN, gelsolin. The red arrow indicates an increase or decrease in the substance next to it, up for an increase, down for a decrease. Figure created by BioRender.

The K238 site of NEDD8-activating enzyme E1 regulatory subunit (NAE1) is its primary crotonylation modification site. Studies have confirmed this by mass spectrometry analysis and found that this site is highly preserved across species (Fig. 2). NAE1, as a subunit of E1-like ubiquitin-activating enzymes, is mainly involved in the Neddylation modification of proteins [13, 14]. Crotonylation modification of the K238 site enhances the Neddylation activity of NAE1. Specifically, K238cr promotes the binding of NAE1 to ubiquitin-like modifier activating enzyme 3 (UBA3), thereby enhancing the catalytic activity of NAE1. This improved activity allows NAE1 to more efficiently attach NEDD8 to its target proteins, which regulates the function of these target proteins [15]. The crotonylation modification of NAE1 K238cr directly affects the Neddylation modification of its downstream target protein, gelsolin (GSN). The principle of action is that K238cr promotes the Neddylation of GSN, which enhances the protein stability and expression level of GSN. GSN is an essential actin-binding protein whose primary function is to regulate the dynamic organization of actin filaments and to promote the depolymerization of actin by cutting off and capping the tips of actin filaments. When the expression and stability of GSN increase, its actin severing activity is also enhanced, leading to unfavorable cytoskeleton remodeling [16]. There is a significant role of biotinylated modification of NAE1 K238cr in CVDs, especially in cardiac hypertrophy. Cardiac hypertrophy is a common cardiac disease characterized by an increased size of cardiomyocytes, cardiac fibrosis, and a progressive decline in cardiac function. It has been shown that the level of crotonylation modification at the K238 locus of NAE1 was significantly up-regulated in a mouse model of cardiac hypertrophy and in patients. By promoting Neddylation and protein stability of GSN, NAE1 K238cr enhances the actin-severing activity of GSN, leading to unfavorable remodeling of the cardiomyocyte cytoskeleton, which in turn contributes to the onset and progression of pathologic cardiac hypertrophy [17, 18]. Furthermore, NAE1 K238R (lysine to arginine mutation) knock-in mice exhibit attenuated cardiac hypertrophy and improved cardiac function, whereas NAE1 K238Q (lysine to glutamine mutation) knock-in mice exhibit increased pathological hypertrophic responses and cardiac dysfunction [15].

Crotonylation modification of NAE1 K238cr has an important impact on the development and progression of CVDs such as myocardial hypertrophy by enhancing its Neddylation activity, which regulates the Neddylation and function of the downstream target protein GSN. However, it is important to note that studies on NAE1 K238cr crotonylation modification and its mechanism of action in CVDs are still in the stage of research. Translating these findings into clinical applications requires further in-depth studies and clinical trials.

**Fig. 3.**
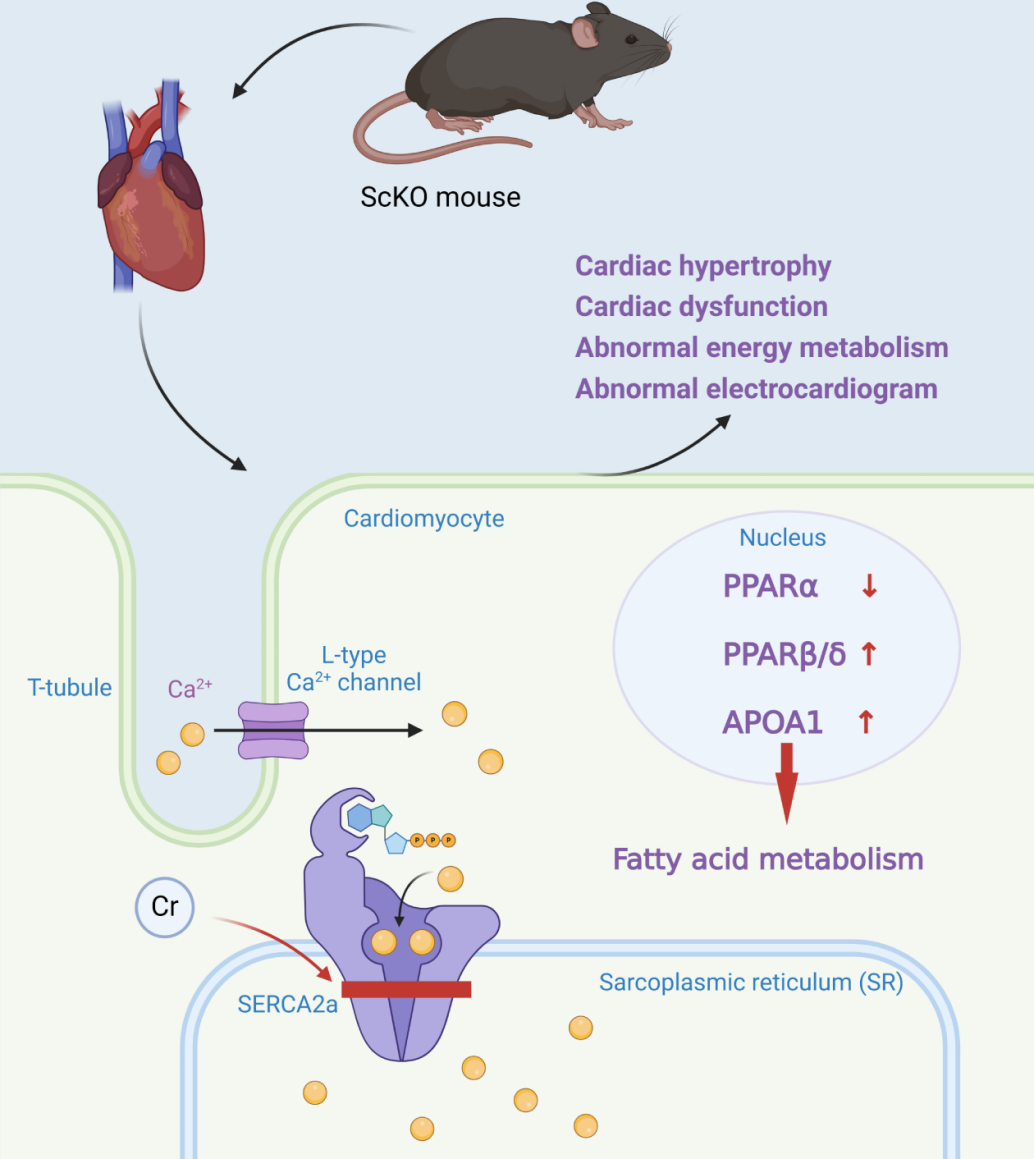
**Work model for SIRT1 regulation in ScKO mice**. SIRT1 knockout can increase the Kcr level of SERCA2a and reduce its activity. It also impacts the expression of proteins in the PPAR pathway, causing abnormal fatty acid metabolism. These changes can result in cardiac hypertrophy, cardiac dysfunction, abnormal energy metabolism, and abnormal electrocardiograms. Schematic created by the authors. Kcr, histone lysine crotonylation; SERCA2a, Sarcoplasmic/Endoplasmic Reticulum Calcium ATPase 2a; PPAR, peroxisome proliferator-activated receptor; SIRT1, slent information regulator type 1; ScKO, srt1 cardiac-specific knockout. The small red arrow indicates an increase or decrease in the substance next to it, up for an increase and down for a decrease. Figure created by BioRender.

Sarcoplasmic/Endoplasmic Reticulum Calcium ATPase 2a (SERCA2a) is a calcium ATPase mainly found in the sarcoplasmic reticulum membrane of cardiomyocytes (Fig. 3). Its principal function is to pump calcium ions (Ca^2+^) from the cytoplasm into the sarcoplasmic reticulum by providing energy through ATP hydrolysis, thus reducing the calcium concentration in the cytoplasm [19]. This process is essential for cardiomyocyte diastole, as cardiomyocyte contraction is triggered by an increase in the concentration of calcium ions in the cytoplasm. SERCA2a, by recycling calcium ions, helps cardiomyocytes to return to a resting state in preparation for the next contraction [20]. The lysine residue K120 of SERCA2a undergoes a crotonoylation modification. This modification alters the structure and function of SERCA2a, and the crotonylation modification leads to a decrease in the binding energy of SERCA2a to ATP, which reduces the enzymatic activity of SERCA2a. SERCA2a is less efficient at recycling calcium ions, leading to impaired diastolic function in cardiomyocytes. Sirtuin 1 (SIRT1) knockout resulted in increased levels of crotonylation of SERCA2a. It affected the expression of proteins related to the peroxisome proliferator-activated receptor (PPAR) signaling pathway, which regulates fatty acid and energy metabolism [21]. SIRT1 knockout mice exhibit reduced expression of PPARα and elevated expression of PPARβ/δ and Apolipoprotein A1 (APOA1), suggesting abnormal energy metabolism in cardiomyocytes [22].

As a result of reduced SERCA2a activity, the diastolic function of cardiomyocytes is impaired, leading to a decrease in the contractile function of the heart, which in turn results in cardiac dysfunction. The excitability and conductivity of cardiomyocytes are regulated by calcium ion concentration. The abnormal function of SERCA2a leads to the mismatch of intracellular calcium ion concentration regulation, which increases the risk of arrhythmias. In addition, studies have also found that SIRT1 knockout mice developed cardiac hypertrophy, which may be the result of their being under an increased load for an extended period in an attempt to maintain cardiac function, which ultimately increases their size. The SERCA2a gene was introduced into cardiomyocytes by genetic engineering techniques to improve the expression level of SERCA2a and thereby enhance its function. This approach has been shown to be beneficial in animal experiments; for example, in a heart failure model, SERCA2a gene therapy mediated by adenoviral vectors, improved cardiomyocyte systolic and diastolic functions and enhanced myocardial contractility. However, gene therapy still faces many challenges in clinical application, such as the safety of viral vectors, long-term gene expression stability, and the immune response.

### 2.2 Non-Cardiomyocytes

Ischemic heart disease (IHD) is a cardiovascular disease which results in myocardial dysfunction due to chronic myocardial ischemia. There are many mechanisms by which ischemic heart disease occurs, one of which is due to smooth muscle cell phenotypic transformation, resulting in vascular remodeling. Vascular smooth muscle cells (VSMCs) undergo phenotypic transformation during vascular injury [23]. VSMC phenotypic remodeling can be regulated by platelet-derived growth factor-BB (PDGF-BB), and crotonylation of non-histone proteins may be involved in this process [24]. Acute myocardial ischemia/reperfusion (I/R) injury also contributes to CVDs, which tends to result in extensive crotonylation of some proteins. These proteins are generally associated with cardiomyocyte contraction. Kcr at mitochondria-specific sites can protect cardiomyocytes from isoprenaline-induced apoptosis by downregulating BNIP3-mediated mitochondrial autophagy and preventing mitochondrial depolarization [25]. Therefore, regulating cardiac Kcr by I/R injury may be a new target for IHD therapy.

## 3. Lactylation

### 3.1 Cardiomyocytes

Lactylation was first reported by Liberti and Locasale [26] at the University of Chicago in 2019. Protein lactylation, a novel metabolic-epigenetic regulatory mechanism, plays a multidimensional role in the pathophysiological progression of CVDs. α-Myosin Heavy Chain (α-MHC) monoidal interactions with Tropomyosin (Titin) play significant roles in the normal contractile function of the heart. The K1897 site of α-MHC is the key site for its lactylation modification, and the lactylation and delactylation of α-MHC K1897 determine its binding and detachment from Titin. The lactylation of α-MHCK 1897 regulates the interaction of α-MHC with Titin. Decreasing the lactylation of α-MHCK 1897 exacerbates heart failure. Excessive lactate efflux and depletion in cardiomyocytes can lead to reduced lactate concentration. This phenomenon is a key factor in the lactation of α-MHC K1897 and the attenuation of the interaction between α-MHC and Titin during myocardial injury [27]. During myocardial injury, lactate levels in cardiomyocytes significantly decrease due to excessive exocytosis of intracellular lactate, which results from the upregulation of monocarboxylic acid transporter 4 (MCT4) expression. The primary function of MCT4, a key member of the MCT family, is to regulate intracellular lactate efflux [28, 29]. In cardiomyocytes, the expression level of MCT4 is usually low under normal conditions, and its expression is significantly up-regulated if the myocardium is injured [30]. In addition, the increased consumption of lactate during myocardial injury may also be responsible for the marked decrease in lactate levels in cardiomyocytes. Lactate consumption is almost tripled in failing hearts with left ventricular ejection fraction below 40% [31]. There is a suggestion that MCT4 inhibitors can restore the lactate level of α-MHC K1897 and the interaction between α-MHC and Titin, which has an ameliorating effect on heart failure [32].

Under normal and heart failure conditions, the acetylation levels of K1897 are significantly different. Acetylation of K1897 enhances the interaction between α-MHC and Titin, which maintains the stability of the sarcomere and the normal contractile function of cardiomyocytes, and allows for normal myocardial contractility under various loading conditions. In heart failure patients and in animal models, the K1897 acetylation level of α-MHC is reduced, weakening its interaction with Titin [27]. This change leads to impaired contractile function of cardiomyocytes and further aggravates heart failure. By increasing the concentration of lactate or inhibiting lactate efflux, the acetylation modification of α-MHC can be restored, thereby enhancing its interaction with Titin and improving the function of cardiomyocytes.

### 3.2 Non-Cardiomyocytes

In post-myocardial infarction macrophages, H3K18la accelerates post-injury cardiac microenvironment remodeling by recruiting histone acetyltransferase general control non-depressible 5 (GCN5) to the promoter region of reparative genes (e.g., IL-10, vascular endothelial growth factor (VEGF)), activating anti-inflammatory and pro-angiogenic pathways [33]. In contrast, lactonization of Snail1 in endothelial cells, promotes endothelial-mesenchymal transition (EndoMT) by enhancing the TGF-β signaling pathway, exacerbating myocardial fibrosis resulting in cardiac damage [34, 35]. In regards to non-histone modifications, acetylation of fatty acid synthase fatty acid synthase (FASN) K673 inhibits its enzymatic activity. It reduces lipid synthesis, which may be protective by inhibiting lipid accumulation in atherosclerotic plaques [36, 37]. In addition, N-myc downstream regulatory gene family member 3 (NDRG 3) is affected by intracellular lactate concentration. Increased intracellular lactate concentration leads to stabilization of NDRG 3 via lactate binding and subsequent activation of downstream signaling cascades [38], the result of which is to drive the conversion of vascular smooth muscle cell VSMCs to a synthetic phenotype and promote plaque formation. The bidirectional regulatory properties of lactonization (e.g., pro-repair versus pro-fibrosis) highlight its spatiotemporal specificity across cell types and disease stages, suggesting that targeting the dynamic balance of lactonization may be a novel strategy for intervening in CVDs. In the future, the interaction of lactonization with other metabolic-epigenetic networks needs to be further analyzed to develop precise therapeutic tools.

## 4. Succinylation

Lysine succinylation was first reported in 2011 by Zhang *et al*. [39] at the University of Chicago, who discovered several succinylation binding sites in *E. coli*. In 2012, a study confirmed the widespread occurrence of succinylation modification in eukaryotic cells. By inducing mutations in modified residues, it was initially shown that lysine succinylation can alter both the structure and function of histones [40]. Succinylation primarily occurs on lysine residues of non-histone proteins (such as mitochondrial proteins) and histones. Succinylation, a significant post-translational modification, involves the covalent attachment of a succinyl group to a lysine residue via enzymatic or non-enzymatic processes. Given its involvement in nearly all biological processes in living organisms, succinylation is highly conserved.

### Cardiomyocytes

The desuccinylases SIRT (sirtuin, SIRT5) and SIRT7 are present in eukaryotes. Research has revealed that in mice models with acute myocardial infarction, the expression of hepatic SIRT5 is up-regulated. Researchers created mice models with liver-specific overexpression of SIRT5 and wild-type mice, and then induced myocardial ischemia to explore the mechanism for limiting acute cardiac ischemia. The results indicated that in mice with overexpression of liver SIRT5, the areas of myocardial infarction and fibrosis were significantly smaller than those in wild-type mice. Nevertheless, the secretion of fibroblast growth factor 21 into the circulation was enhanced. It was thus hypothesized that in the myocardial ischemia model, SIRT5 exerted its cardioprotective effects through a liver-heart cross-talk mechanism [41]. Another study probed the impact of the PTMs Mitsugumin53 (MG53 for short), which are associated with acute myocardial infarction and membrane repair, on cardiomyocyte apoptosis and inflammation [42]. It was demonstrated that the succinylation of MG53K130 is promoted by lysine acetyltransferase 3B, and is inhibited by SIRT7. In addition, the succinylation site of MG53 coincides with its ubiquitination site. The succinylation of MG53 also facilitates its ubiquitination, thereby triggering the degradation of MG53 and reducing its protein level. This in turn exacerbates hypoxia/reoxygenation-induced cardiomyocyte injury. As a result, researchers may take advantage of the desuccinylation function of SIRT7 to inhibit the ubiquitination of MG53 and upregulate its protein level, to achieve selective targeted therapy for myocardial infarction [43].

Currently, research on specific protein targets of succinylation in CVDs is inadequate. Elucidating the succinylation mechanisms of these targets and their crosstalk or synergy with other PTMs can enhance our understanding of the pathophysiology of these diseases and provide new theoretical interventions for clinical therapy. Regulators of the SIRT protein family, including inhibitors and activators, are important for future research. Targeted regulators, as potential clinical agents, will need continued exploration, development, and validation.

## 5. Conclusion

This review provides an overview of three common PTMs and their roles in various CVDs. PTMs are prevalent in the development and progression of CVD, but their pathogenesis and pathologic roles are currently unclear. Further study of the mechanism of action of PTMs on CVD can broaden our understanding of CVD and improve the level of treatment. Since CVD has a profound impact on people’s lives, it has become an important focus of research.

In recent years, PTMs have been closely related to the occurrence of CVDs and have gradually become an important area of medical research. More than 450 unique patterns of PTMs have been identified, and this study focuses on the most common types. Most PTMs are reversible and control the state of body functions by controlling the state of the cells. These types of PTMs are not only regulated to protect against physiological damage but also act synergistically in several ways to ensure that cells can respond quickly and accurately to external stimuli. Unlike transcriptional translation, protein translation is a dynamic process rapidly engaged in the maintenance and functions of barriers. PTMs occur primarily through their involvement in cardiovascular signaling pathways, mitochondrial oxidative stress, and cardiomyocyte apoptosis. It is well known that CVDs are highly prevalent and harmful, and their prevalence increases with age. Therefore, new strategies for the treatment of CVDs are being investigated. Continued research in this area will improve patients’ quality of life by preventing and mitigating the effects of cardiovascular diseases.
